# Recent Advances in Solid-State Hydrogen Storage Based on Metal Hydrides and Nanoporous Carbon Materials

**DOI:** 10.3390/nano16171049

**Published:** 2026-08-22

**Authors:** Bakhytzhan Lesbayev, Moldir Auyelkhankyzy, Gaukhar Ustayeva, Nurgali Rakhymzhan, Aidos Tolynbekov, Ayazhan Zhamash, Meruyert Nazhipkyzy

**Affiliations:** 1 Faculty of Chemistry and Chemical Technology, Farabi University, 71 Al-Farabi Ave., 050040 Almaty, Kazakhstan; lesbayev@mail.ru (B.L.); meruert82@mail.ru (M.N.); 2 Institute of Combustion Problems, 172 Bogenbay Batyr, 050012 Almaty, Kazakhstanaasszhamashaass@gmail.com (A.Z.)

**Keywords:** hydrogen storage, compressed hydrogen, liquefied hydrogen, intermetallic compounds, metal hydrides, nanoporous carbon, spillover, adsorption, desorption

## Abstract

Hydrogen is considered one of the most promising energy carriers for sustainable and carbon-neutral energy systems. However, the large-scale deployment of hydrogen technologies is limited by the lack of efficient, safe, and cost-effective hydrogen storage methods. This review examines current hydrogen storage technologies and the physical and chemical mechanisms underlying hydrogen adsorption. Traditional storage approaches, including compressed gas and liquid hydrogen, are briefly analyzed with respect to their advantages, limitations, safety concerns, and energy requirements. Special focus is given to solid-state hydrogen storage systems based on metal hydrides, which offer high storage capacities and enhanced operational safety. Recent advances in intermetallic hydrides, magnesium-based materials and complex hydrides are discussed, along with challenges related to thermodynamic stability, sorption kinetics, thermal management, and cycling durability. This review also highlights recent developments in nanoporous carbon materials and the role of the hydrogen spillover mechanism in improving adsorption performance. Experimental studies reporting hydrogen adsorption capacities above 7 wt.% and up to 11.2 wt.% are analyzed. Based on the reviewed literature, key research directions are identified for optimizing the adsorption properties of advanced materials and accelerating the development of efficient and sustainable hydrogen storage technologies for future energy applications.

## 1. Introduction

A reliable energy supply is a key factor in sustainable socioeconomic development. However, the global energy sector’s continued dependence on fossil fuels has significant environmental consequences, including increased greenhouse gas emissions, pollution, and heightened climate risks. Consequently, interest in low-carbon energy technologies has grown significantly in recent decades, among which hydrogen holds a special place due to its high energy density and significant potential for integration with renewable energy sources. The development of hydrogen energy is considered by many countries as a key area of the energy transition. National hydrogen strategies shape technological development directions, as well as regulatory and economic mechanisms for the creation of new chains for the production, transportation, storage, and use of hydrogen. Large-scale programs aimed at deploying hydrogen technologies to decarbonize the energy sector are already being implemented in developed economies. Among the most significant initiatives are those of the European Union, the United States, and Japan, which aim to develop technologies for the production, storage, and use of hydrogen, create the necessary infrastructure, and stimulate the hydrogen energy market [1,2,3].

A vital role is also played by the international program “Global Programme for Hydrogen in Industry (GPHI)”, implemented under the auspices of the United Nations Industrial Development Organization (UNIDO) and aimed at supporting developing countries and countries with economies in transition in the field of implementation of hydrogen technologies [4].

As part of its climate policy, the European Union plans to expand green hydrogen production and achieve a significant reduction in greenhouse gas emissions by 2030 [5]. The Republic of Kazakhstan has adopted a Concept for the Development of Hydrogen Energy until 2030, which views hydrogen as a key element in the transition to a low-carbon economy and the decarbonization of industry and the transport sector [6].

Interest in hydrogen as a clean fuel stems from its versatility: it can be used as a fuel, an energy carrier, and a means of storing excess energy. Hydrogen is particularly important in the context of the growing share of renewable energy sources, as it enables the long-term storage of excess energy generated during periods of low demand by solar, wind, nuclear power plants, and other low-carbon sources. The most promising method for large-scale production of clean hydrogen is water electrolysis using electricity generated from renewable sources [7].

Compared to traditional fuels, hydrogen has the highest energy density per unit mass. However, due to its extremely low bulk density at normal ambient temperature and pressure, hydrogen has a low energy density per unit volume, creating significant challenges in developing storage systems. Therefore, one of the key factors hindering the widespread use of hydrogen as an alternative fuel remains the lack of safe, cost-effective, and technologically reliable storage and transportation systems. One of the main challenges in developing storage and transportation systems is the negative impact of hydrogen on the properties of the structural materials used to construct storage tanks. According to the US (United States) Department of Energy (DOE) targets [8], for large-scale implementation of hydrogen technologies, promising hydrogen storage systems must meet the following criteria: system hydrogen capacity of at least 7.5 wt.%; release temperature in the range of 60–120 °C; normal operating pressure of 35 or 70 bar. Currently, three main approaches to hydrogen storage are used: as compressed gas; as liquid hydrogen in a cryogenic environment; as solid materials through chemical binding of hydrogen or physical adsorption [9,10].

Table 1 presents the average comparative characteristics of the three principal hydrogen storage approaches, including gravimetric and volumetric storage capacities, operating conditions, kinetic performance, and safety aspects. The values are synthesized from an analysis of publicly available literature and represent generalized ranges intended for comparative assessment rather than the performance of specific materials or storage systems.

The analysis shows that none of the technologies under consideration simultaneously provides high gravimetric and volumetric capacity, fast kinetic characteristics, low operating pressures and a high level of safety. Therefore, with the development of nanotechnology, the development of hydrogen storage materials using nanostructured solid-state carriers is of scientific interest. These carriers, due to their unique properties, can combine all the requirements in a single hydrogen storage system. Such materials include porous materials, nanoporous carbon, and metal–organic frameworks (MOFs) [11]; clathrates, amides, and zeolites [12,13,14,15,16]; metal hydrides and intermetallic compounds [17,18,19]. These materials can store hydrogen through chemical or physical adsorption mechanisms [20]. The development of lightweight and affordable solid-state hydrogen storage systems based on nanostructured materials, characterized by high capacity and fast kinetics of adsorption and desorption processes, can contribute to solving the problem of practical application of hydrogen fuel cells in transport and other mobile energy applications.

Over the past five years, nanostructured materials have become a key research area in hydrogen storage due to the fundamental changes in the mechanisms of hydrogen-matter interactions observed at the nanoscale. Unlike bulk materials, nanostructures are characterized by a high specific surface area, shortened diffusion path lengths, a large number of interphase boundaries, and an increased concentration of structural defects, which significantly impacts both the kinetics and thermodynamics of hydrogen adsorption and absorption processes [21]. As a result, the activation energy for hydrogen dissociation and diffusion decreases, the rate of hydrogenation increases, and the ability to control equilibrium storage characteristics through structural engineering becomes possible.

Surface phenomena and spatial confinement effects (nanoconfinement) occurring in nanopores, nanoparticles, and thin-film systems play a special role. A high proportion of surface atoms, altered coordination environments, defect engineering, catalytic interphase boundaries, and strong interactions between the support and the active phase make it possible to modify the hydrogen binding energy, increase the reversibility of sorption processes, and improve storage kinetics [22,23]. For metal hydrides, an additional advantage of nanostructuring is the dependence of the hydride formation enthalpy and equilibrium pressure on particle size, opening opportunities for targeted control of the thermodynamic properties of materials.

Current research is characterized by a shift from studying individual classes of materials to designing multifunctional nanostructured systems. The most rapidly developing materials are nanoporous carbon materials, graphene and its derivatives, MOFs and covalent organic frameworks (COFs), nanocrystalline metal hydrides, high-entropy alloys, and hybrid composites that combine physical adsorption, chemical bonding, and catalytic hydrogen storage mechanisms [24]. At the same time, computational modeling and machine learning methods are gaining widespread acceptance, accelerating the search for new nanomaterials with optimal storage properties [25]. In recent years, computational methods have evolved from auxiliary research tools into one of the principal approaches for the development of next-generation hydrogen storage materials. Whereas earlier studies primarily relied on density functional theory (DFT) calculations and classical thermodynamic models, current research increasingly employs integrated computational frameworks that combine DFT, molecular dynamics (MD), grand canonical Monte Carlo (GCMC) simulations, phase-field modeling, finite element analysis (FEA), and multiscale simulations [26]. Such integrated approaches enable the description of hydrogen adsorption, diffusion, hydride formation, and heat transfer across multiple spatial and temporal scales, providing a more comprehensive understanding of the mechanisms governing hydrogen storage.

At the same time, machine learning (ML) and artificial intelligence (AI) have emerged as rapidly expanding research paradigms, enabling the identification of complex relationships between chemical composition, crystal structure, and functional material properties [27]. The combination of large experimental and computational databases with high-throughput screening (HTS) has substantially accelerated the discovery of promising metal hydrides, high-entropy alloys, MOFs, COFs, and nanoporous carbon materials with optimized hydrogen storage performance, while significantly reducing the need for costly and time-consuming experimental investigations [28]. In parallel, the Calculation of Phase Diagrams (CALPHAD) methodology is increasingly being employed to predict phase stability, thermodynamic properties, and hydride formation behavior in complex multicomponent systems [29].

The combined application of CALPHAD, DFT, and machine learning algorithms provides a powerful framework for the rational design of hydrogen storage materials with tailored thermodynamic characteristics, including hydride formation enthalpy, equilibrium pressure, and reversible hydrogen storage capacity. Consequently, the concept of data-driven materials discovery, based on the integration of computational modeling, artificial intelligence, and automated analysis of large-scale materials datasets, is now widely recognized as one of the most promising strategies for accelerating the development of high-performance hydrogen storage materials. In this context, artificial intelligence is increasingly viewed not only as a computational tool but also as an integrative framework that connects empirical observations, theoretical modeling, and engineering design, thereby enabling more efficient and systematic development of next-generation hydrogen storage systems. The combination of nanoscale effects, rational structural design, and digital methods in materials development defines the modern scientific paradigm for creating highly efficient hydrogen storage systems and is driving growing interest in this area of research.

This article provides a concise overview of the current state of conventional hydrogen storage technologies, including compressed hydrogen storage and liquid hydrogen systems. Attention is given to hydrogen storage in metal hydrides and nanoporous carbon materials derived from plant waste. The advantages, challenges, and prospects of these materials as efficient and sustainable hydrogen storage media are discussed in detail. Electrochemical hydrogen storage and clathrate hydrate-based storage technologies are not covered.

## 2. Hydrogen Storage

### 2.1. High-Pressure Hydrogen Storage

One of the most common methods for storing hydrogen at ambient temperatures is the use of high-pressure cylinders filled with compressed gas, which are used in both industry and the transportation sector. The main advantages of this method include high filling speed, which is especially important for mobile applications; the availability of a well-developed infrastructure and compatibility with existing commercial equipment; and proven maintenance, storage, and transportation processes. However, this method has several significant drawbacks, including low volumetric energy density (even at 700 bar, the volumetric energy density is 3–4 times lower than that of gasoline); the significant weight of cylinders, especially metal ones; the high cost of composite cylinders; the risk of hydrogen leakage; and the potential for explosion in the event of mechanical damage or improper operation. Currently, various types of cylinders are used for high-pressure hydrogen storage, differing in design, materials, and technical characteristics.

Type I gas cylinders (steel and aluminum) have been used since the late 19th century to store various gases, including hydrogen, and are designed for operating pressures of up to 300 bar. However, due to their significant mass (from 50 to 500 kg), they are characterized by a low gravimetric storage capacity, which limits their use in mobile applications. Therefore, such cylinders are used primarily for stationary hydrogen storage, for example, at hydrogen filling stations [30,31]. In the 1990s, developments in carbon fiber production technologies made it possible to manufacture Type II gas cylinders (metal body with carbon fiber composite reinforcement), which, compared to Type I gas cylinders, have higher strength characteristics at a lower mass and ensure reliable operation of hydrogen storage systems at pressures of up to 300 bar [32].

In the early 2000s, Type III composite cylinders with a metal liner became widespread, allowing hydrogen storage at pressures up to 350–700 bar. A significant weight reduction compared to Type I and II gas cylinders significantly expanded their potential for use in mobile applications [33,34].

During the same period, the development and implementation of type IV gas cylinders began [35,36], characterized by the presence of an internal polymer liner and full composite reinforcement. Due to the use of a polymer liner and carbon fiber reinforcement, type IV gas cylinders are significantly lighter than previous analogs, provide a high level of tightness, resistance to mechanical damage and corrosion, and can withstand pressures of up to 700 bar. Since the 2010s, type IV gas cylinders have become widely used in hydrogen vehicles and containerized hydrogen storage and transportation systems. Despite the advantages of type IV gas cylinders, their use is associated with certain disadvantages, including the vulnerability of the polymer liner to damage during a sharp decrease in pressure, low thermal conductivity, leading to a significant increase in temperature inside the cylinder during rapid hydrogen filling, limited thermal stability under sudden temperature changes, and high production costs [37,38,39,40,41].

Type V cylinders, which are fully composite structures without an inner liner, are currently the subject of intensive research and development. Owing to their exceptionally low weight and high strength, they are considered a promising solution for hydrogen storage in aviation, aerospace, and transportation applications, where minimizing weight and maximizing volumetric efficiency are critical. The first Type V cylinder prototypes were developed in 2014 by Composite Technology Development for aerospace applications. For V-type cylinders, further technological improvements and cost reductions in their production are currently required [42,43].

**Figure 1:**
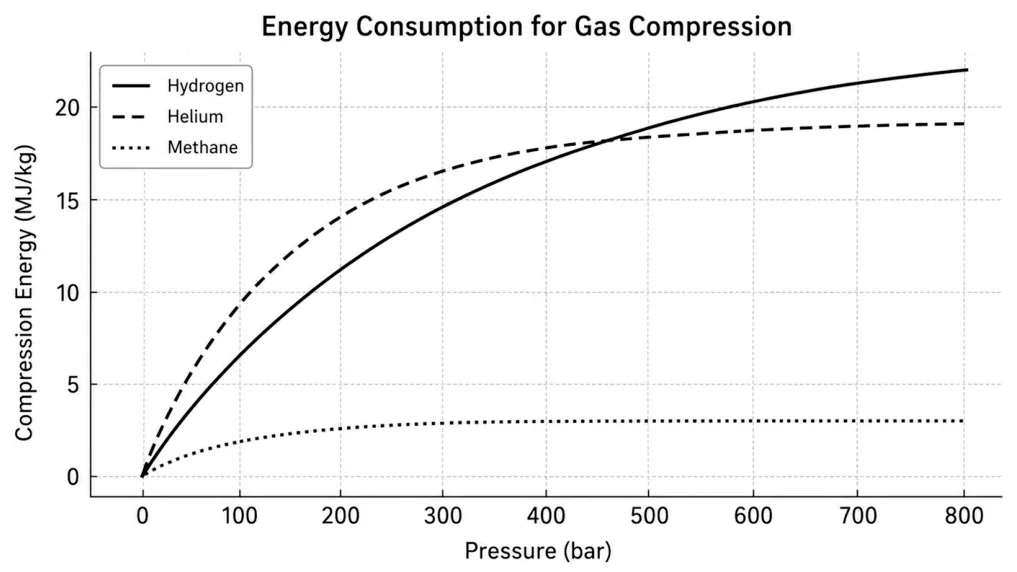
Comparative analysis of the dependence of compression energy on pressure for various gases.

Despite significant progress in the development of hydrogen storage systems as compressed gas under high pressure, this approach still faces several limitations for widespread use in the transportation sector. One of the main reasons is the low volumetric energy density of hydrogen, even when using modern composite cylinders designed for pressures up to 700 bar. An analysis of the dependence of specific energy consumption on compression pressure for various gases (Figure 1) shows that compressing hydrogen requires approximately eight times more energy than methane, significantly impacting the cost-effectiveness of compressed hydrogen storage technologies.

Recent advances in hydrogen compression technologies have therefore focused on improving the thermodynamic efficiency of the compression process while reducing thermal losses. Liquid piston hydrogen compressors have attracted considerable attention as a promising alternative to conventional mechanical compressors because the liquid piston simultaneously compresses the gas and enhances heat removal, resulting in a compression process closer to isothermal conditions. Recent numerical studies [44,45] based on coupled computational fluid dynamics (CFD) and heat transfer simulations demonstrated that optimization of the compression chamber geometry, including its radius and height, can significantly increase compressor productivity while limiting hydrogen temperature rise. Furthermore, the thermal behavior of the compression chamber has been shown to depend strongly on wall thickness, with thicker walls reducing transient temperature fluctuations due to their higher thermal inertia, thereby decreasing thermal loading and improving the stability of repeated compression cycles. These advances contribute to lower compression energy consumption, improved system reliability, and higher overall efficiency of hydrogen storage and refueling infrastructure, highlighting the importance of compressor design optimization alongside improvements in storage materials.

Prospects for the further development of high-pressure hydrogen storage systems are linked to the need to improve their safety and energy efficiency by increasing the specific density of stored hydrogen. An important area of research is the creation of new high-strength composite materials for cylinders, characterized by low weight and high resistance to cyclic loads and corrosion. Current research in the field of compressed hydrogen storage focuses not only on the classification of types I–V cylinders but also on addressing key engineering challenges that determine their durability and safety. A primary focus is on improving composite materials based on carbon fiber and epoxy or thermoplastic matrices, which can increase specific strength, reduce structural weight, and extend the service life of high-pressure vessels. At the same time, new polymer liners and protective coatings with reduced hydrogen permeability are being actively developed, reducing leaks and preventing material degradation during long-term operation [46].

Attention should be paid to the development of engineering solutions aimed at improving the operational safety of high-pressure cylinders using modern sensor monitoring technologies. Fatigue failure of composite cylinders under repeated loading cycles remains an important problem. Modern research is aimed at studying the accumulation of microdamage, delamination, crack formation in the polymer liner, as well as the development of defects in the composite shell under the influence of cyclic pressure, temperature fluctuations and hydrogen penetration processes [47]. To improve reliability, numerical modeling and residual life assessment methods are widely used [48].

In recent years, structural health monitoring (SHM) technologies have been intensively developed. They are based on the use of built-in fiber-optic sensors, acoustic emission, piezoelectric and strain gauge sensors, providing continuous monitoring of the stress–strain state of composite cylinders in real time [49,50].

The combined application of artificial intelligence and machine learning techniques offers expanded capabilities that will enable real-time analysis of sensor data to detect early signs of damage, predict the remaining service life of hydrogen storage systems, and implement the concept of predictive maintenance [51]. AI systems can simultaneously process and analyze data from hydrogen sensors, acoustic detectors, thermal imaging cameras, and pressure monitoring systems, which will improve the efficiency of leak detection with exceptional accuracy and speed [52]. Such approaches are considered one of the most promising areas for the creation of intelligent and safe next-generation hydrogen storage systems, and AI acts not only as a computational tool, but also as a connecting mechanism that unites the empirical, theoretical, and practical areas of hydrogen storage systems.

### 2.2. Liquefied Hydrogen Storage Technology

In 1898, James Dewar first liquefied hydrogen at temperatures below −252.9 °C and demonstrated that liquefied hydrogen occupies 800 times less volume than hydrogen at ambient conditions. For comparison, when compressed to 700 bar, hydrogen’s density increases significantly; however, liquid hydrogen has a higher bulk density than compressed hydrogen at a given pressure. Unlike storing hydrogen under high pressure, storing hydrogen in liquid form, due to its significant volume reduction, is one of the most effective methods for compact hydrogen storage, especially for applications requiring the transportation or storage of large volumes of hydrogen. Significant technological progress in this area was achieved in the mid-20th century with the development of rocket technology. Since the 1980s, research into the use of liquefied hydrogen in transportation applications has been actively developing. The main advantages of liquefied hydrogen storage compared to compressed hydrogen storage include the ability to achieve relatively high volumetric (≈70 kg/m^3^) and energy (≈2.4 kWh/L) densities during storage and transportation. However, liquefied hydrogen storage technology has several significant limitations. Maintaining hydrogen in a liquid state requires temperatures below −253 °C, necessitating the use of a highly efficient thermal insulation system and specialized cryogenic equipment. Furthermore, the liquefaction process is characterized by high energy consumption, accounting for 30–40% of the energy content of the resulting liquid hydrogen. An additional problem is hydrogen loss due to evaporation during long-term storage, which requires the installation of continuous pressure monitoring systems and maintenance of safety systems. These factors remain the main obstacles to the widespread adoption of liquid hydrogen storage technologies. Nevertheless, research into improving cryogenic storage systems continues, and current advances in this area are presented in [53,54].

Promising areas for further research aimed at improving the efficiency and economic feasibility of liquefied hydrogen storage primarily revolve around the development of next-generation integrated cryogenic systems. A key aspect is the creation of highly effective composite thermal insulation systems for cryogenic tanks that combine low thermal conductivity, mechanical stability, and durability under cyclic thermal loads. Attention should be paid to hierarchical multilayer insulation materials (including vacuum powder and aerogel structures), which can significantly reduce heat gain and, consequently, the rate of hydrogen evaporation.

In addition to the problems of storing liquid hydrogen associated with high energy costs for liquefaction, evaporative losses (boil-off losses) and the need to use highly efficient cryogenic thermal insulation, there is currently an active search for new technologies aimed at increasing the efficiency and operational reliability of cryogenic systems. One of the most promising areas in this area is cryogenically compressed hydrogen storage (cryo-compressed hydrogen), which combines low storage temperatures (20–80 K) with high pressure (up to 250–350 bar) [55,56]. This approach provides a higher volumetric storage density compared to compressed gas, significantly reduces the evaporation rate and allows for an increase in the range of vehicles, especially in heavy road transport, rail equipment and aviation.

One of the fundamental problems with storing liquefied hydrogen remains the loss caused by inevitable evaporation (boil-off). In this regard, a promising direction is the development of closed-loop energy systems in which evaporating hydrogen is considered not as a loss, but as an additional energy resource. It is advisable to integrate fuel cells into the storage system, which convert part of the evaporated hydrogen into electrical energy. The resulting energy can be used to power active cooling systems, as well as to operate hydrogen re-liquefaction units. In recent years, much research has been devoted to the development of integrated cryogenic fuel systems in which the storage tank, heat exchange equipment, evaporator, pressure control system, and fuel cells are combined into a single energy system [57]. Such integration is considered one of the key areas of development of hydrogen aviation, space technology and heavy freight transport.

Another rapidly developing area is zero-boil-off storage technologies based on the use of highly efficient multilayer vacuum thermal insulation, active cryocoolers, cold recovery, and programmable heat flow control [58,59]. Modern Zero Boil-Off systems can eliminate hydrogen emissions during long-term storage, which is especially important for aerospace applications, marine transportation of liquid hydrogen, and mobile power plants. These technologies are currently considered one of the most promising areas for the development of cryogenic hydrogen infrastructure due to their combination of high storage density, minimal fuel loss, and increased operational safety.

### 2.3. Thermodynamic Principles and Theoretical Basis of Hydrogen Adsorption and Absorption

The performance of hydrogen storage systems is governed by the fundamental thermodynamic principles controlling the processes of physical adsorption in porous materials and chemical absorption in metal hydride systems. In both cases, the equilibrium between the gaseous and bound states of hydrogen is determined by the Gibbs free energy change, which depends on the enthalpy and entropy of the process. Physical adsorption is characterized by relatively weak van der Waals interactions, whereas hydrogen absorption by intermetallic compounds involves the formation of chemical bonds and the development of a hydride phase, accompanied by significantly larger thermal effects.

One of the principal equations describing the temperature dependence of the equilibrium hydrogen pressure in metal hydride systems is the Van’t Hoff equation:

(1) $$\ln P=\frac{\Delta H}{RT}-\frac{\Delta S}{R}$$

where

*P* is the equilibrium hydrogen pressure;

Δ*H* is the enthalpy of hydride formation (J mol^−1^);

Δ*S* is the entropy of hydride formation (J mol^−1^K^−1^);

*R* is the universal gas constant;

*T* is the absolute temperature (K).

This equation establishes a linear relationship between ln *P* and 1/*T*, allowing the thermodynamic parameters of hydride formation to be determined experimentally. The enthalpy Δ*H* characterizes the strength of the chemical bonding between hydrogen and the metal and determines the amount of heat released during hydriding and absorbed during dehydriding. The entropy Δ*S* reflects the change in the degree of order as molecular hydrogen transitions from the gaseous phase to the chemically bound state.

To describe the equilibrium adsorption of gases in microporous materials, the Dubinin–Astakhov (DA) model is widely employed. This model is based on the Theory of Volume Filling of Micropores (TVFM). Unlike the Langmuir and BET models, the DA approach treats adsorption as a process of volumetric filling of micropores driven by the adsorption potential. The DA equation is expressed as

(2) $$W=W_{0}\exp\left[-{\left(\frac{A}{E}\right)}^{n}\right],$$

where

*W* is the volume of adsorbate filling the micropores at a given temperature and pressure (cm^3^g^−1^ or mmol g^−1^);

*W*_0_ is the limiting micropore volume (maximum adsorption capacity) (cm^3^g^−1^);

*A* is the adsorption potential (J mol^−1^);

*E* is the characteristic adsorption energy (J mol^−1^);

*n* is the structural heterogeneity parameter of the micropores (empirical constant).

The adsorption potential is defined as

(3) $$A=RT\ln\left(\frac{P_{0}}{P}\right),$$

where

*R* is the universal gas constant;

*T* is the absolute temperature (K);

*P*_0_ is the saturated vapor pressure of the adsorbate (for supercritical gases such as hydrogen, a virtual saturation pressure or characteristic pressure is typically used);

*P* is the equilibrium hydrogen pressure.

The Dubinin–Astakhov model enables accurate description of experimental hydrogen adsorption isotherms, determination of micropore volume, calculation of the characteristic adsorption energy, evaluation of temperature effects, and prediction of hydrogen storage capacity.

One of the most important thermodynamic parameters describing the interaction energy between hydrogen molecules and the adsorbent surface is the isosteric heat of adsorption ($Q_{st}$), which is determined from the Clausius–Clapeyron equation:

(4) $$Q_{st}=-R\frac{ln\left(P_{2}/P_{1}\right)}{\left(1/T_{2}-1/T_{1}\right)}$$

where

$Q_{st}$ is the isosteric heat of adsorption (kJ mol^−1^);

*R* is the universal gas constant;

*P*_1_ and *P*_2_ are the pressures corresponding to the same adsorption loading at temperatures *T*_1_ and *T*_2_;

*T*_1_ and *T*_2_ are the absolute temperatures.

Unlike the enthalpy of hydride formation, the isosteric heat of adsorption characterizes the interaction energy between hydrogen molecules and the adsorbent surface and typically does not exceed several kilojoules per mole. Consequently, physical adsorption exhibits high reversibility and rapid kinetics but generally requires cryogenic or reduced temperatures to achieve high hydrogen storage capacities.

The enthalpy of hydride formation (Δ*H*) and the isosteric heat of adsorption ($Q_{st}$), are key thermodynamic parameters that determine the operating temperature range, equilibrium pressure, energy efficiency, and overall performance of hydrogen storage systems. For adsorption materials, optimal $Q_{st}$ values are generally in the range of 10–15 kJ mol^−1^, providing sufficiently strong hydrogen adsorption while maintaining reversible desorption. For metal hydrides, the enthalpy of hydride formation typically falls within 20–40 kJ mol^−1^, enabling high volumetric hydrogen storage densities but requiring heat input during hydrogen release.

Thus, the Dubinin–Astakhov model, the Clausius–Clapeyron equation, and the Van’t Hoff equation constitute the theoretical foundation for the thermodynamic analysis of hydrogen storage processes. The Dubinin–Astakhov model describes the mechanism of micropore filling and the equilibrium adsorption capacity, the Clausius–Clapeyron equation quantifies the energetics of hydrogen interaction with the adsorbent surface, and the Van’t Hoff equation characterizes the thermodynamics of metal hydride formation and decomposition. Together, these models provide a quantitative framework for describing hydrogen adsorption and absorption processes and serve as the basis for the development of advanced hydrogen storage materials with improved performance.

## 3. Hydrogen Storage Technology Using Metal Hydrides

Chemical hydrogen storage methods are based on its reversible interaction with solids to form hydrides. This approach is considered a promising way to create safe and efficient hydrogen storage systems. From an applied perspective, metal hydrides are conventionally divided into two groups: “low-temperature” and “high-temperature”. This division is based on the operational requirements of hydrogen storage systems and is not a strictly scientific classification. Low-temperature metal hydrides include those for which the equilibrium hydrogen pressure exceeds atmospheric pressure at temperatures up to 100 °C. This group includes hydrides based on the intermetallic compounds AB_5_, AB_2_, and AB, as well as pseudobinary hydrides of vanadium-based alloys and the Ti-Cr system. High-temperature metal hydrides are primarily magnesium-based alloys and intermetallic compounds. The main advantage of metal hydride hydrogen storage systems is their high safety. However, a significant drawback of most hydrides with high hydrogen storage capacity is the elevated temperatures required for hydrogenation and dehydrogenation processes, which limits their use in mobile applications. Significantly higher gravimetric capacities for hydrogen are characteristic of complex hydrides [60]. In particular, the theoretical capacity of LiBH_4_ is approximately 18.5 wt.% H_2_ [61], which makes this material one of the most promising in terms of specific energy capacity. However, its practical application is also limited by high temperatures (above 400 °C) and pressures (above 350 bar), hydrogenation and dehydrogenation processes, and it is also characterized by low chemical stability to the effects of moisture and oxygen. An exception are hydrides based on intermetallic compounds, which can function effectively at room temperature, moderate pressures, and are resistant to impurities, but their hydrogen capacity is small—on the order of 1.5–2 wt.% [62].

In addition to gravimetric and volumetric hydrogen capacity, an important factor determining the applicability of metal hydrides in hydrogen storage systems are their thermodynamic characteristics, which determine the equilibrium conditions of interaction in the metal-hydrogen system [63,64,65]. In this context, of particular interest are the developments of metal hydrides based on high-entropy alloys capable of functioning under moderate conditions of temperatures from 0 to 100 °C and pressures from 1 to 40 bar, which can be found in [66,67,68,69,70]. A significant limitation of the practical application of most metal hydrides is the slow kinetics of hydrogen adsorption and desorption processes, caused by limited heat transfer and contamination with secondary impurities [71,72,73]. Improvement of the kinetics of hydrogen adsorption and desorption processes by metal hydrides can be achieved through product purity, modification of the chemical composition by alloying, and the use of catalysts. Modern advances in this area are discussed in detail in review papers [74,75,76].

Due to its widespread availability, low cost, and high capacity, magnesium is considered one of the most promising hydride-forming materials for hydrogen storage systems. Metal hydrides based on it have a high theoretical hydrogen storage capacity—up to 7.6% by weight—and can operate at moderate pressures. However, the practical application of magnesium-based hydrides is limited by high operating temperatures (300–400 °C) and the slow kinetics of hydrogenation and dehydrogenation processes. Therefore, research aimed at improving these characteristics is a topic of active scientific interest. Table 2 provides a comparative overview of the properties of MgH_2_-based composites containing different dopants. According to Table 2, authors [77] can decrease the onset temperature for dehydrogenation until 160 °C.

In research work [78], hydride–intermetallic composites MgH_2_ + X wt.% FeTi (X = 10, 30, 50) were synthesized by controlled mechanical grinding and the effect of FeTi content, as well as additional nickel alloying, on hydrogen desorption processes was investigated. The results are summarized in Table 3.

It was found that an increase in the FeTi proportion leads to a linear decrease in the hydrogen desorption peak temperature and the activation energy of MgH_2_ desorption, indicating an improvement in the kinetics of the composite dehydrogenation process. The incorporation of 5 wt.% nanosized nickel was found to further enhance the catalytic activity, resulting in a decrease in the hydrogen desorption peak temperature to below 300 °C for composites containing 10 and 30 wt.% FeTi. The greatest effect is observed for the MgH_2_–10 wt.% FeTi composite; the introduction of 5 wt.% Ni provides the greatest reduction in dehydrogenation temperature (≈88 °C). At 30 wt.% FeTi, the reduction is approximately 68 °C, while at 50 wt.% FeTi, the position of the main hydrogen desorption peak remains virtually unchanged. The authors report that under these conditions a second desorption peak appears and for the MgH_2_ + 50 wt.% FeTi + 5 wt.% n-Ni composite a decrease in the temperature of the second desorption peak by approximately 26 °C is observed. This indicates that at high FeTi content, the catalytic activity of the system, determined solely by the intermetallic compound, and the additional effect of Ni becomes limited.

In work [79], zirconium titanate (ZrTiO_4_) was synthesized by a hydrothermal method and incorporation into MgH_2_ in an amount of 7 wt.%. The results showed that the incorporation of ZrTiO_4_ reduces the initial dehydrogenation temperature of MgH_2_ by 70 °C compared to pure MgH_2_ subjected to similar mechanical grinding (248.8 °C) and significantly improves the kinetics of both hydrogen sorption and desorption. The MgH_2_—7 wt.% ZrTiO_4_ composites can release 6.3 wt.% H_2_ at 300 °C in 5 min and absorbing up to 5.5 wt.% H_2_ at 125 °C in 10 min, which indicates the high efficiency of this approach for improving the hydrogen characteristics of MgH_2_.

In work [84], the effect of alloying magnesium hydride with the multicomponent TiCrNbH_x_ alloy was investigated. The addition of 20 wt.% TiCrNbH ensured hydrogen absorption already at room temperature, and the dehydrogenation onset temperature was 163 °C. At a temperature of 230 °C, this composite released up to 5.8 wt.% hydrogen in 700 s, maintaining a reversible hydrogen capacity of 4.98 wt.% after 100 sorption–desorption cycles. In work [80], the catalytic effect of hafnium tetrachloride (HfCl_4_) on the thermodynamic and kinetic parameters of the dehydrogenation and hydrogenation processes of MgH_2_ was considered. It was found that the addition of 15 wt.% HfCl_4_ lowers the onset temperature of MgH_2_ decomposition by 75 °C compared to unmodified mechanically ground MgH_2_. Doping with 15 wt.% HfCl_4_ significantly improves the hydrogen kinetics. The resulting composite can adsorb about 5.5 wt.% and desorbing ~4.5 wt.% hydrogen within 5 min, while unmodified MgH_2_ under similar conditions absorbs only ~4.0 wt.% and releases only ~0.5 wt.% hydrogen. According to calculations using the Kissinger method, the activation energy of dehydrogenation decreases from 167.0 kJ/mol (pure MgH_2_) to 102.0 kJ/mol, which confirms the high catalytic efficiency of HfCl_4_.

A significant disadvantage of metal hydrides is the decrease in hydrogen capacity with an increase in the number of adsorption and desorption cycles, which is caused by the physical and chemical degradation of the material [86]. This effect can be partially mitigated by doping with transition metals. In [87], the effect of iron (Fe) alloying on the hydrogen storage properties of a titanium-chromium-molybdenum alloy (Ti–Cr–Mo) was investigated. The resulting composite alloy of the composition Ti_40_Cr_48_Mo_10_Fe_2_ with a body-centered cubic lattice demonstrates a decrease in the enthalpy of dehydrogenation to 32.4 kJ/mol and the ability to hydrogenate at a temperature of 303 K and dehydrogenate at 333 K, achieving a hydrogen capacity of 2.59 wt.%. This indicates an improvement in the thermodynamic characteristics of the system. The authors of [87] also found that the decrease in capacity during cycling is associated with hydrogen-induced phase transformations, the accumulation of internal stresses, and the formation of irreversible titanium hydrides, which hinder the diffusion and release of hydrogen. The results obtained demonstrate the promise of Fe alloying as an approach to increased cyclic stability and durability of intermetallic hydrogen storage systems.

In work [88], it was shown that the combined modification of MgH_2_ with the Ni–MoS_2_ hybrid catalyst significantly improves the kinetics of hydrogen adsorption/desorption processes, as well as the cyclic stability of the material. The introduction of 10 wt.% Ni–MoS_2_ by ball milling allows the initial temperature of MgH_2_ dehydrogenation to be reduced to 198 °C, with the reverse hydrogenation process already beginning at 50 °C. The resulting composite exhibits high hydrogen evolution kinetics, releasing up to 6.1 wt.% H_2_ in 10 min at 300 °C due to a decrease in the activation energy of dehydrogenation from 151 to 85 kJ/mol. It was found that during the interaction of nickel with MgH_2_, the Mg_2_NiH_0.3_ phase is formed, which acts as an active center for hydrogen transfer, while the layered structure of MoS_2_ ensures efficient dispersion of MgH_2_ and nickel nanoparticles. The synergistic action of the Mg_2_NiH_0.3_ and MoS_2_ phases ensures high cyclic stability, with the reversible capacity maintained at 6.1 wt% for up to 50 cycles. The authors also note that the addition of Ni–MoS_2_ has virtually no effect on the overall thermodynamic properties of MgH_2_ but does promote local weakening of the Mg–H bonds, facilitating hydrogen desorption processes.

The review [89] identifies three key approaches to improving the properties of Mg-containing hydrides: doping, catalytic modification, and nanoengineering, each aimed at simultaneously optimizing the thermodynamics and kinetics of hydrogenation-dehydrogenation processes. Doping allows regulation of thermodynamic stability by weakening Mg–H bonds due to changes in the electronic structure and distortion of the crystal lattice. At the same time, it promotes acceleration of the kinetics due to grain refinement and an increase in the density of phase boundaries. Catalytic modification using transition metals, their oxides, and MXenes leads to the formation of active centers that facilitate the dissociation of molecular hydrogen, its migration, and interphase transfer. This reduces the energy barriers to reactions, promotes destabilization of the hydride phase, and the formation of a developed network of diffusion paths. In turn, nanoengineering approaches make it possible to further reduce the thermodynamic stability of the hydride due to surface energy effects, shorten the length of diffusion paths, and increase the concentration of active centers. Taken together, the strategies discussed in the review form a comprehensive toolkit for increasing the efficiency of hydrogen storage in magnesium-based hydrides.

Nanosized metal hydride particles are of particular interest, as their size decreases to the nanoscale, reducing the atomic packing density and increasing the proportion of atoms located on the nanoparticle surface, which improves diffusion and the kinetics of hydrogen sorption–desorption. Review articles [90,91] detail advances in the synthesis, structure and properties, and theoretical studies of nanostructured metal hydrides. The goal of these studies is to optimize the thermodynamic and kinetic reactions of hydrogen storage. One promising direction is the creation of composites by combining metal hydride nanoparticles with porous materials to prevent agglomeration.

In work [92], a strategy for producing nanostructured materials based on the Mg–Y system was proposed, based on the adsorption of pure metal particles on the surface followed by low-temperature heat treatment. It was found that the formation of a nanoscale structure leads to a significant improvement in the hydrogen sorption properties of the material. The resulting nanomaterial has an increased hydrogen capacity (~4.5 wt.%) compared to the cast alloy, as well as a lower dehydrogenation temperature. The authors attribute the improvement in properties to microstructural changes and a reduction in the characteristic dimensions of structural elements to nanoscale, which facilitates the acceleration of hydrogen adsorption and desorption processes.

One of the effective tools for predicting the behavior of hydride materials under various conditions (temperature, pressure, alloy composition, particle size, etc.) is numerical modeling. In work [93], quantum chemical modeling using density functional theory (DFT) was performed, which made it possible to establish the fundamental mechanisms of the effect of alloying with transition metals on the properties of MgH_2_. It was shown that modeling allows one to predict changes in the heat of formation, desorption temperature, and stability of the hydride phase without complex experimental studies. Based on an analysis of charge transfer, differences in electronegativity, and the density of electron states, it was established that a decrease in the thermodynamic stability of MgH_2_ is associated with a weakening of the Mg–H bond. The authors showed that niobium (Nb) is a promising alloying element, providing an optimal combination of the heat of formation and desorption temperature. Thus, numerical modeling is an effective tool for the targeted search for alloying components to optimize the kinetic and thermodynamic characteristics of metal hydrides.

In work [94], the effects of unconventional doping of metal hydrides with the NH_4_^+^ cation were examined using simulation. The simulations revealed mechanisms of electron density redistribution and dihydrogen bond formation that are difficult or impossible to observe experimentally. The authors demonstrated that the weak interaction of doped NH_4_^+^ with hydrogen atoms contributes to an increase in hydrogen storage capacity and a decrease in the thermal stability of the hydrides. This work demonstrates that modern computational methods can not only explain the observed effects but also predict new approaches to modifying hydrogen storage materials, including poorly studied types of doping.

In work [95], modeling is used to describe the processes of hydrogen physical adsorption in slit nanopores of carbon materials and boron nitride. The developed thermodynamic model considers the quantum effects of hydrogen molecule behavior within the confined pore volume and utilizes an extended equation of state for hydrogen, which correctly describes the system over a wide range of temperatures and pressures. The model reproduces experimental data on hydrogen storage in nanoporous carbon materials and enables prediction of optimal pore sizes, operating pressures, and temperatures to achieve hydrogen storage targets established by the U.S. DOE.

The results of studies [93,94,95] demonstrate that modern modeling methods are a vital tool for developing promising hydrogen storage materials. Quantum chemical calculations allow us to establish the relationship between the electronic structure and thermodynamic properties of hydrides. Thermodynamic modeling of adsorption processes helps predict the optimal architecture of porous materials and the conditions for their effective operation. The use of computational methods significantly reduces the volume of expensive experimental studies and accelerates the search for materials with desired hydrogen storage characteristics.

In conclusion of this section, it should be noted that metal hydride hydrogen storage systems, due to the strong chemical binding of hydrogen in a solid matrix, provide one of the safest methods of hydrogen storage. Despite their high potential for adsorption capacity, most hydrides are characterized by unfavorable thermodynamic parameters of hydrogenation/dehydrogenation processes, which limit their practical applicability. An exception is intermetallic hydrides, which can function under moderate conditions; however, their hydrogen capacity does not exceed ~1.5–2 wt.%. Theoretically, high hydrogen capacity (~18 wt.%) can be provided by complex hydrides such as LiBH_4_; however, their practical application is limited by unsatisfactory thermodynamic and kinetic parameters. Among promising materials, Mg-based hydrides are of particular interest, possessing high theoretical capacity (up to 7.6 wt.%) at moderate pressures. However, their use is also limited by high operating temperatures (300–400 °C) and slow kinetics of hydrogenation/dehydrogenation processes.

Considering the above, the primary objectives in developing highly efficient metal hydrides for hydrogen storage remain research related to the targeted control of their thermodynamic and kinetic characteristics. Priority areas for future research include:

− Tuning the thermodynamics of hydride formation through alloying and the formation of intermetallic phases with specified equilibrium pressures, ensuring an operating temperature range close to ambient conditions;
− Accelerating sorption/desorption kinetics by catalysts and reducing particle size to the nanoscale;
− Developing composite systems (metal hydride + nanoporous carbon/graphene/heat-conducting matrices) that ensure efficient heat and mass transfer, as well as increased stability and resistance to degradation, agglomeration, and active surface area loss during repeated hydrogenation/dehydrogenation cycles;
− Comprehensive modeling that establishes the relationship between the composition, structure, and properties of materials to create and optimize the specified performance characteristics of hydrogen storage materials.

## 4. Nanoporous Carbon in Hydrogen Storage Systems

**Figure 2:**
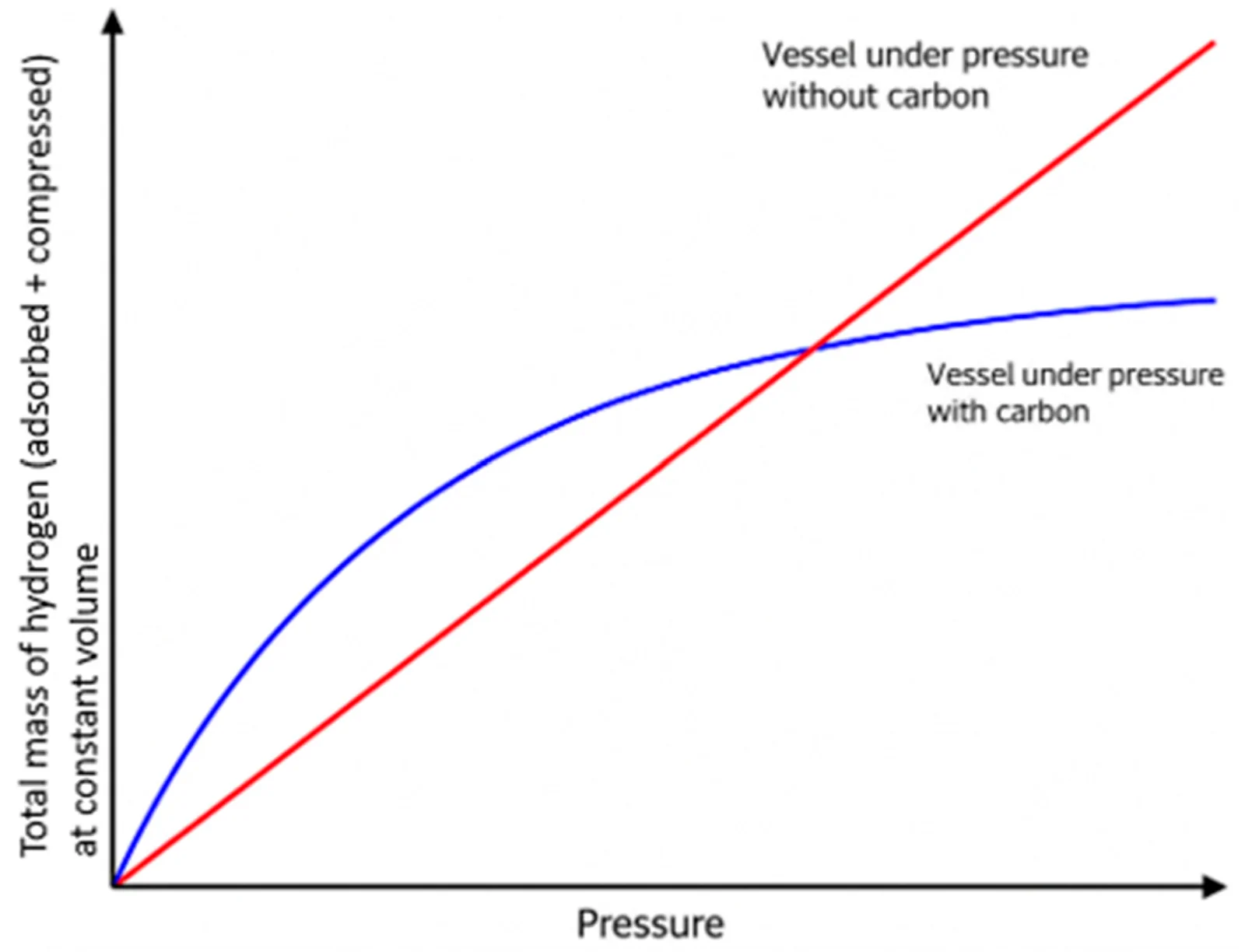
Schematic comparison of the mass of hydrogen stored in a high-pressure vessel without an adsorbent and in a vessel filled with a porous carbon material. The difference between the curves characterizes the increase in storage capacity due to hydrogen adsorption.

In physical adsorption, hydrogen molecules are adsorbed on the surface of a porous material due to van der Waals forces, resulting in an increased hydrogen molecule concentration at the gas/solid interface compared to its bulk concentration in the gas phase. This phenomenon opens the possibility of increasing the hydrogen storage capacity in pressurized cylinders by incorporating porous materials with a high specific surface area (Figure 2).

The graph in Figure 2 demonstrates that the amount of adsorbed hydrogen increases with increasing pressure. The mass of hydrogen contained in an empty cylinder increases almost linearly with increasing pressure, whereas for a cylinder filled with a porous material, the relationship is nonlinear. This indicates that, within a certain pressure range, the presence of an adsorbent ensures the storage of an amount of hydrogen exceeding its content in the free volume under the same thermodynamic conditions. Thus, excess adsorption is the amount of hydrogen absorbed by the pore walls of a porous material, in addition to the amount of hydrogen that occupies the volume of the adsorbent under the same temperature and pressure conditions.

The adsorption properties of a porous solid depend primarily on its pore size. To achieve high gravimetric hydrogen capacity, it is necessary not only to increase the total specific surface area but also to optimize the pore size distribution. According to the IUPAC classification, pore sizes are divided into micropores (<2 nm), mesopores (2 to 50 nm), and macropores (>50 nm). Micropores, in turn, are divided into supermicropores (0.7–2.0 nm) and ultramicropores (<0.7 nm). Above a certain critical point, hydrogen adsorption occurs predominantly in micropores, and its density exceeds the density of the unadsorbed bulk phase in meso- and macropores or voids. Consequently, hydrogen adsorption increases with increasing accessible micropore surface area. Porous materials that adsorb gaseous substances by physical adsorption are characterized by a low enthalpy of adsorption; for hydrogen, it is approximately 4 kJ/mol at 77 K, i.e., the adsorption efficiency increases with decreasing temperature to cryogenic levels. The explosion hazard of the “microporous adsorbent-hydrogen” system is eliminated by the high degree of hydrogen dispersion by the microporous structure of the adsorbent, which prevents the formation of an explosive concentration of hydrogen. Thus, structural regulation of the porosity of the material plays a decisive role in improving its sorption properties and safety. A thermodynamic model of hydrogen storage (the Mills–Younglove model) in slit pores, which is applied to carbon and nanoporous materials, was presented in [96]. A distinctive feature of the model is a new equation of state for hydrogen, which considers the quantum effects of molecules in the confining potential of slit pores in the temperature range of 77–300 K and pressure of 0–1000 bar. The model predicts that to meet the US DOE’s hydrogen storage targets, the optimal nanopore width should be 5.6 Å at 77 K regardless of pressure, and about 6 Å at 300 K at pressures above 10 bar.

Significant advances in the use of nanoporous materials for hydrogen storage have been discussed in detail in several review papers, with particular attention paid to the role of binding energy, specific surface area, and the influence of pore shape and size on hydrogen adsorption and desorption processes [97,98,99,100,101,102]. Key characteristics of nanoporous carbon as a promising material for hydrogen storage include high specific surface area, stable framework structure, the possibility of large-scale production, and fast kinetics of hydrogen adsorption and desorption processes. In an experimental study [103], studies were conducted to determine the upper limit for hydrogen storage at 77 K on activated carbons obtained by chemical activation of anthracite. The uniqueness of this study lies in the fact that nanoporous carbon was obtained in independent experiments at the Wroclaw University of Technology (Poland) and the Jean Lamour Institute (France), and the results obtained were confirmed in three independent laboratories using volumetric and gravimetric instruments. In [104], it was shown that the maximum theoretical specific surface area of a graphene sheet is 2630 m^2^/g. Based on these data, the authors of [103] calculated that the theoretical limiting sorption capacity of graphene for hydrogen is 6.8 wt.%. The authors of [103], on the obtained activated carbon with Brunauer–Emmett–Teller (BET) surface area of 3220 m^2^/g, achieved hydrogen adsorption of 6.4 wt.% at 77 K and 4 MPa, which was designated as the upper limit of hydrogen adsorption by nanoporous carbon.

**Figure 3:**
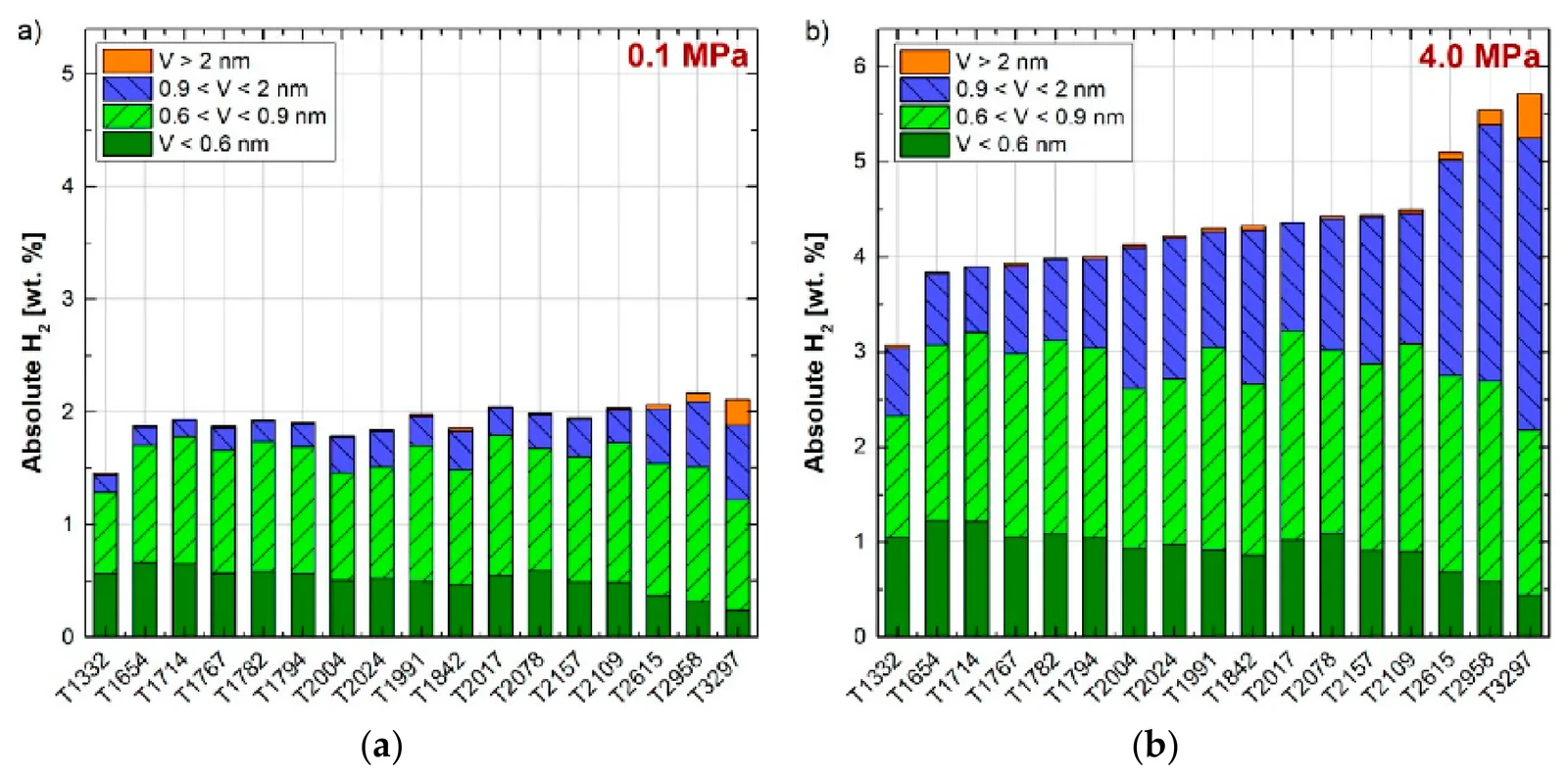
Contribution of each pore size range to hydrogen adsorption at: (**a**) 0.1 MPa; (**b**) 4.0 MPa [105].

The authors [105], based on experimental data on hydrogen adsorption for 17 samples of activated carbons [103] and using the mathematical model proposed in the work [106], calculated the contribution of each range of pore sizes to hydrogen adsorption at 0.1 MPa Figure 3a and 4.0 MPa Figure 3b.

**Figure 4:**
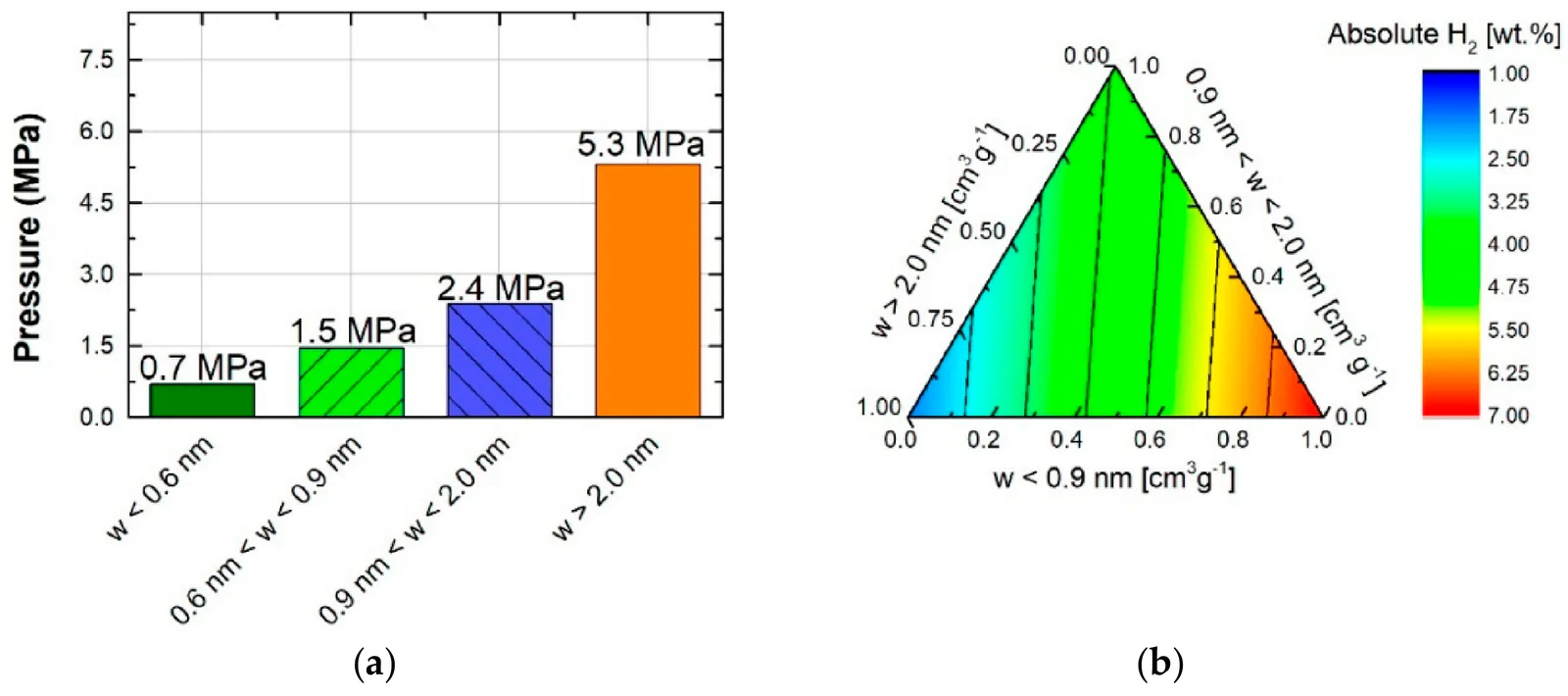
The pressure required to achieve 95% of the maximum adsorption density for each pore size range (**a**); the absolute H_2_ uptake as a function of pore volume at 77 K and 4.0 MPa (**b**) [105].

Based on the obtained results of the study, the authors [105] concluded that the contribution of ultramicropores with a size of about 0.6 nm to the total hydrogen storage capacity at 77 K will be effective at a pressure of about 1 MPa, and to achieve a higher capacity of adsorbed hydrogen, the presence of pores wider than 0.6 nm and pressures above 3 MPa are necessary. The authors also calculated the pressure required to achieve 95% of the maximum hydrogen adsorption density for each pore size range; the results are presented in Figure 4a. For narrow pores and ultramicropores, this pressure is achieved at 0.6 MPa, and for mesopores larger than 2.0 nm, 5.4 MPa is required. To identify the effect of pore size distribution at a pressure of 4 MPa, the authors also performed calculations for a hypothetical material with a total pore volume of 1.0 cm^3^/g. The results are presented in Figure 4b.

According to the calculations, a carbon material consisting of pores smaller than 0.9 nm can adsorb more than 6 wt.%, while a material consisting of mesopores will adsorb less than 2 wt.%. The results show that the hydrogen storage capacity is determined not by the total surface area or the total pore volume, but by the pore size distribution. The most effective are narrow micropores (<0.6 nm) and ultramicropores (0.6–0.9 nm), providing the maximum density of adsorbed hydrogen even at relatively low pressures. Thus, the authors of the reviewed works [105] suggested the existence of an upper limit of hydrogen adsorption by nanoporous carbon materials (~6.4 wt.% at 77 K). The studies discussed below show that the supposed limit is not a fundamental limitation.

Of particular interest is the use of biomass waste as a feedstock for producing nanoporous carbon. Due to their availability, diversity, renewability, chemical compositional versatility, the ability to control textural characteristics, and relatively low cost, these materials are considered among the most promising precursors for creating hydrogen adsorbents. The study [107] examines current advances in the production of porous carbon materials from biomass and analyzes their potential for use in hydrogen storage technologies. The results of the study demonstrate that structural tuning of the porosity of carbon materials, depending on the production methods and choice of the starting bio-raw material, plays a key role in enhancing their sorption properties. Two independent studies [108,109] provide illustrative examples in this area, in which activated carbon obtained from different components of loblolly pine demonstrated significantly different hydrogen storage characteristics.

In work [108], activated carbon was synthesized from loblolly pinecones. Pre-washed pinecones were crushed and heated at 453 K for 5 h to remove residual moisture. The resulting cone powder was subjected to thermochemical activation with KOH at different mass ratios of precursor and activator (0.5:1, 1:1, and 3:1). Among the obtained samples, the material synthesized at a 1:1 ratio of pinecone powder to KOH had the best textural characteristics. This material was characterized by a specific surface area of 1173 m^2^/g and a micropore volume of 0.383 cm^3^/g. In a study of hydrogen adsorption at a temperature of 77 K, it demonstrated the best storage capacity: 1.6 wt.% at a pressure of 1 bar and 5.25 wt.% at a pressure of 80 bar. In the study [109], nanoporous carbon was also synthesized from loblolly pine. The feedstock was subjected to hydrothermal carbonization at temperatures of 180 °C, 220 °C, and 260 °C, after which the resulting carbonizates were activated with KOH at temperatures of 700 °C, 800 °C, and 900 °C. The mass ratio of KOH to carbonizate was varied within the ranges of 2:1, 3:1, and 4:1. The best textural characteristics were achieved for the sample obtained at a hydrothermal carbonization temperature of 260 °C and subsequent chemical activation at 800 °C with a KOH:carbonizate ratio of 4:1. This material has a specific surface area of 3666 m^2^/g, a total pore volume of 1.560 cm^3^/g, and a micropore volume of 1.32 cm^3^/g. The maximum adsorption capacity of the resulting nanoporous carbon reached 10.2 wt% at a temperature of 77 K and a pressure of 55 bar. The authors demonstrated that increasing the specific surface area by approximately 3 times and the micropore volume by 3.5 times led to an approximately twofold increase in the hydrogen adsorption capacity at a significantly lower pressure. Thus, the results of the studies indicate that both the nature of the feedstock and the thermochemical processing conditions have a decisive influence on the formation of the porous structure and, consequently, on the efficiency of hydrogen storage in nanoporous carbon materials.

Rice husk is one of the most common byproducts of rice processing, with hundreds of millions of tons produced annually. Due to its combination of organic components (cellulose, hemicellulose, and lignin) and significant silica content, it represents a promising precursor for producing nanoporous carbon with a hierarchical structure consisting of micro-, meso-, and macropores. The review [110] provides a detailed discussion of methods for synthesizing nanoporous carbon materials based on rice husk, as well as the environmental, catalytic, and energy aspects of their practical application. The resulting materials are also being actively studied as hydrogen adsorbents.

In work [111], the influence of the mass ratio of NaOH to rice husk on the formation of textural characteristics of nanoporous carbon obtained by thermochemical activation was systematically studied. Preliminary carbonization of the raw material was carried out at 400 °C, after which the sample was activated with NaOH at different reagent ratios at a temperature of 800 °C for 1–2 h. It was found that optimization of the activation conditions ensures the formation of a highly developed porous structure with a specific surface area of up to 3969 m^2^/g and a total pore volume of 2.03 cm^3^/g. The obtained material demonstrates high efficiency in cryogenic hydrogen adsorption processes, achieving a storage capacity of 7.7 wt.% at 77 K and a pressure of 1.2 MPa.

In work [112], the thermochemical activation of rice husk using KOH was investigated, with an emphasis on studying the effect of cooling conditions of the activated carbon material on its adsorption characteristics with respect to hydrogen. It was shown that the cooling mode has a significant effect on the distribution of pore sizes with a virtually unchanged specific surface area. It was found that rapid cooling promotes the formation of a more developed microporous structure and provides a maximum hydrogen storage capacity of 7.2 wt.% at 77 K, whereas with slow cooling under ambient conditions, this figure decreases to 5.5 wt.%. The authors concluded that the cooling conditions of the nanoporous carbon obtained in the process of thermochemical activation have a decisive influence on the development of the microporous structure and, therefore, on the adsorption properties of the resulting material.

In work [113], an approach based on preliminary mechanical activation of rice husk before the carbonization stage was proposed. As a result, nanoporous carbon was obtained with a specific surface area calculated by the BET method of 2713 m^2^/g, a specific surface area of micropores by the DR method of 3099 m^2^/g and a total pore volume calculated by the Barrett–Joyner–Halenda (BJH) method of 1.625 cm^3^/g. The adsorption capacity of the obtained sample for hydrogen was 3.7 wt.% at a temperature of −190° C and a pressure of 9 kg/cm^2^, which was 29.7% higher than the similar figure for nanoporous carbon obtained from rice husk without preliminary mechanical activation. The authors suggested that mechanical activation leads to loosening of the intercellular substance and partial depolymerization of the plant matrix components, increasing the accessibility of the internal structure of the material and ensuring more controlled formation of pores in the processes of carbonization and subsequent thermochemical activation.

In work [114], a combined approach to the synthesis of nanoporous carbon from rice husks was proposed, including preliminary high-temperature enzymatic-osmotic treatment of the raw material. It is assumed that during fermentation, partial destruction of the lignin matrix occurs, which provides access to the amorphous phase of SiO_2_ and promotes its subsequent effective dissolution and removal during the thermochemical activation of KOH. According to the authors, this mechanism leads to the formation of a developed carbon framework with a high degree of porosity. The resulting material is characterized by a specific surface area of 2270 m^2^/g and demonstrates a hydrogen storage capacity of 1.21 wt.% at 25 °C and a pressure of 80 bar, which is among the highest values for nanoporous carbon materials under conditions close to room temperature. Taken together, the presented results confirm the significant potential of rice husks as a renewable precursor to produce highly efficient nanoporous carbon materials intended for use in hydrogen storage systems.

**Figure 5:**
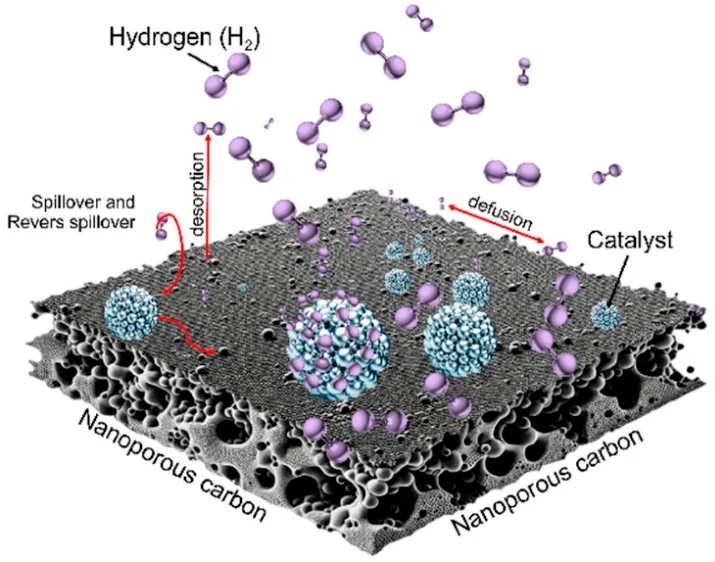
Illustration of the hydrogen transfer mechanism according to the «spillover» mechanism [113].

The paper [115] examines and analyzes a concept for storing hydrogen in porous materials based on the “spillover” mechanism. To implement this mechanism, metal nanoparticles (Pd, Pt, Ni) are introduced into the porous material structure, acting as catalysts for hydrogen dissociation. The resulting hydrogen atoms migrate from the surface of the metal particles into the pores of the porous material, overcoming the relatively low activation energy (<10 kJ/mol). Figure 5 shows an illustration of the hydrogen transfer mechanism.

An advantage of this concept is the possibility of increasing the efficiency of hydrogen storage at temperatures close to room temperature. Experimental studies [116,117,118] have shown that one of the most promising methods for increasing the efficiency of hydrogen storage at moderate temperatures is the creation of carbon nanocomposites modified with palladium in combination with oxygen and nitrogen heteroatoms or various functional groups. A common feature of these studies is the use of catalytically active Pd nanoparticles and a chemically modified carbon matrix, which ensure the efficient dissociation of molecular hydrogen and the subsequent migration of atomic hydrogen to active adsorption centers.

In work [116], it was established that doping activated carbon with oxygen-containing functional groups, and in [117,118], doping graphene structures with nitrogen, promotes an increase in the dispersion of Pd nanoparticles and enhances their interaction with the carbon matrix. This leads to an increase in the reversible hydrogen storage capacity at moderate temperatures and pressures. The highest capacity values were obtained for Pd-decorated nitrogen-containing graphene structures, for which the hydrogen storage capacity reached 1.9 wt.% at 25 °C and a pressure of 2 MPa [118]. For Pd-decorated oxygen-containing activated carbons, the increase in storage capacity was up to 48% compared to unmodified activated carbon [116]. The obtained research results indicate that the efficiency of the “spillover” mechanism is determined not only by the presence of palladium, but also by the chemical state of the carbon matrix surface, the type of functional groups, the level of heteroatomic doping, and the dispersion of palladium nanoparticles. An important conclusion is that with increasing pressure the contribution of the “spillover” mechanism gradually decreases, while the dominant role of the mechanism becomes the physical adsorption of hydrogen in the porous structure of the material [116].

Confirmation of the possibility of increasing the efficiency of hydrogen storage due to the “spillover” mechanism by introducing metallic platinum (Pt) nanoparticles into the structure of nanoporous carbon is presented in the experimental work [119]. In the work [119], nanoporous carbon with a specific surface area of 3155 m^2^/g was obtained from bamboo raw material by hydrothermal carbonization followed by microwave treatment and doping with nitrogen. In the next step, a composite carbon material containing platinum nanoparticles in an amount of 2.87–6.73 wt.% was synthesized by the wet impregnation method. The adsorption properties of the obtained materials with respect to hydrogen were studied in two modes: at a temperature of 77 K and a pressure of up to 1 bar, and at a temperature of 298 K and a pressure of up to 4 MPa. It was found that at 77 K, the adsorption capacity for hydrogen was determined mainly by the parameters of the porous structure and the nitrogen content and was practically independent of the Pt concentration. At 298 K, samples doped with platinum nanoparticles exhibited a significant increase in the hydrogen storage enhancement factor (HSE) to 1.82 compared to the original activated carbon. The authors attribute the observed increase in hydrogen adsorption capacity at 298 K to the “spillover” mechanism, which involves the dissociation of molecular hydrogen on the surface of Pt nanoparticles, followed by the migration of atomic hydrogen into the carbon matrix. Thus, analysis of the presented results demonstrates that combining nitrogen doping, oxygen-containing functional groups, and catalytically active metals capable of effectively dissociating hydrogen molecules into atoms may be a promising strategy for increasing reversible hydrogen storage capacity at temperatures close to room temperature and moderate pressures. However, a more in-depth analysis of empirical data from studies of the “spillover” mechanism reveals that the increase in adsorbed hydrogen mass exhibits significant variability and depends on the nature of the metal, the size and distribution of the nanoparticles, the interfacial contact area, the pore structure, and the measurement technique. In several studies, repeated experiments have yielded effects that are substantially smaller than those originally reported or have failed to reproduce the reported results.

Theoretical calculations using the density functional theory (DFT) method show that the efficiency of the spillover mechanism is determined by the energy barriers to H_2_ dissociation, migration of hydrogen atoms through the metal-carrier interface and their further diffusion over the surface [120,121] predict that by optimizing interfacial contact and creating low migration barriers, a significant increase in the amount of adsorbed hydrogen is possible. At the same time, calculations show that even the formation of small amounts of stable surface hydrides at the phase boundary can lead to an increase in the energy barrier and a decrease in the efficiency of the spillover mechanism by limiting the migration range of atomic hydrogen. Based on the above, it can be concluded that experimental data currently indicate the possibility of the spillover mechanism, but its quantitative contribution to the overall hydrogen storage capacity remains a subject of debate.

One interesting area of research is the use of non-traditional man-made and difficult-to-recycle waste as raw materials to produce functional nanoporous materials with specified structural characteristics. An example is the work [122], in which cigarette filters were used to synthesize nanoporous carbon. The authors investigated the properties of nanoporous carbon obtained from both new and used filters collected after smoking. The feedstock was pre-crushed and subjected to hydrothermal carbonization in an autoclave at a temperature of 250 °C for 2 h. The resulting carbonizate was then activated with KOH at a ratio of 4:1 in a nitrogen atmosphere at temperatures of 600, 700, or 800 °C. The heating rate was 3 °C min, and the duration was 1 h.

The study found that the best textural and sorption properties are achieved using used filters as a precursor, indicating the significant role of their initial chemical composition. The authors attribute the ability to produce nanoporous carbon with a large specific surface area (up to 4300 m^2^/g) and a micropore content of approximately 90% to the presence of alkaline and alkaline earth elements (K, Ca, Na, Mg, etc.) accumulated during filter operation. These elements, along with KOH, promote additional activation of the carbon matrix, ensuring the formation of a developed microporous structure. Simultaneously, hydrothermal carbonization leads to the formation of oxygen-containing functional groups (–COOH, C–OH, O–C), which further modify the surface properties of the material and the adsorption energy profile. Thanks to a combination of high specific surface area, developed microporosity, and surface functionalization, the resulting nanoporous carbon demonstrated a hydrogen storage capacity of 9.4 wt% at −196 °C and 20 bar. As the pressure increased from 30 to 40 bar, the adsorption capacity increased to 10.4 and 11.2 wt%.

This study is significant not only for its practical application in developing highly effective adsorbents but also as a source of fundamental principles governing the mechanisms of porous structure formation in carbon materials. It is demonstrated that the chemical composition of the precursor, particularly the presence of embedded alkali and alkaline earth elements, can act as an internal (in situ) activating factor, enhancing the action of an external activator (KOH) and directing the pore formation process. This allows us to consider the precursor not simply as a carbon source, but as a complex reaction system capable of controlling the development of the material’s texture. The obtained results contribute to the concept of programmable pore formation, according to which control of the structure of nanoporous carbon can be achieved not only by varying activation conditions, but also through targeted selection or modification of the precursor (introduction of catalytically active impurities, preliminary functionalization). This approach opens the possibility of transferring the identified patterns to a wide class of carbon-containing sources (biomass, polymers, industrial waste), ensuring the controlled formation of micropores (<2 nm), which are critical for hydrogen adsorption.

Although a high specific surface area and pore size distribution remain important characteristics of carbon adsorbents, modern research shows that hydrogen storage efficiency is determined by a much broader range of structural factors. A significant role is played by pore connectivity, which ensures efficient transport of hydrogen molecules to internal adsorption centers, as well as hierarchical porosity, in which macro- and mesopores function as transport channels, and micropores serve as the main adsorption centers [123]. This multilevel architecture reduces diffusion limitations, accelerates sorption kinetics, and increases the utilization of the material’s internal surface.

Equally important factors are the density of structural defects, heteroatom doping and surface functionalization [101,124,125]. Crystal lattice defects and heteroatoms alter the electronic structure of carbon, create additional active sites for interaction with hydrogen molecules, and increase the heat of adsorption, which is especially important for increasing capacity at temperatures close to room temperature. Functional groups and surface modification with metal nanoparticles (Pt, Pd, Ni, etc.) also enhance hydrogen interaction with the surface and can improve storage efficiency through the spillover mechanism [116,117,118,119].

In recent years, new classes of carbon materials have been rapidly developed, including graphene and doped graphene, carbon aerogels, and carbide-derived carbons (CDCs) [126,127]. Graphene materials possess a high specific surface area, tunable electronic structure, and the ability to be targeted for doping, while three-dimensional carbon aerogels provide developed hierarchical porosity, low density, and high accessibility of adsorption sites. Carbide-derived carbons are characterized by a narrow micropore distribution and a high degree of pore size control at the atomic level, which allows for optimization of hydrogen adsorption energy. The combination of these approaches is considered one of the most promising avenues for the development of highly efficient carbon materials for next-generation hydrogen storage systems.

The efficiency of hydrogen storage systems is assessed not only by the magnitude of the gravimetric capacity, since their practical application is determined by a set of interrelated operational and engineering characteristics. Along with high mass capacity, volumetric capacity is of great importance, determining the amount of hydrogen that can be stored in the limited volume of the system [128]. For mobile applications (automotive, aviation and rail transport), it is the combination of high gravimetric and volumetric capacity that is one of the main efficiency criteria, which is reflected in the technical requirements of the US Department of Energy (U.S. DOE) [6].

Important criteria include cyclic stability, charge and discharge kinetics, and the thermal conductivity of the material. High cyclic stability ensures capacity retention after multiple hydrogenation-dehydrogenation cycles, while fast kinetics determines the charging time and hydrogen delivery to the consumer. For metal hydride and adsorption systems, thermal conductivity becomes a critical factor, since the sorption and desorption processes are accompanied by significant thermal effects. Increasing thermal conductivity using metal fillers, graphite, graphene, or carbon nanomaterials intensifies heat exchange and significantly reduces the system charging time [128].

In addition to technical characteristics, production cost, environmental impact throughout the life cycle assessment (LCA), and overall system efficiency are of great importance for hydrogen storage systems. When selecting a storage technology, it is necessary to consider not only the properties of the material itself, but also the weight of the housing, thermal insulation, heat exchangers, auxiliary equipment, energy consumption for compressing or liquefying hydrogen, as well as operating costs and the possibility of recycling materials at the end of their service life. Therefore, modern research increasingly uses an integrated engineering approach based on Techno-Economic Analysis (TEA) and LCA, which allows comparing different storage technologies considering their efficiency, safety, cost, and environmental sustainability [25]. This systematic approach remains a necessary condition for the transition from laboratory research to industrial implementation of hydrogen storage technologies.

In conclusion of this section, the following conclusions can be formulated. Nanoporous carbon materials demonstrate high potential for hydrogen storage due to their developed microporous structure and high specific surface area. However, their practical application is limited by the low energy of physical adsorption (~4–6 kJ/mol), necessitating the use of cryogenic temperatures to achieve high adsorption capacities. A promising direction is the targeted optimization of ultramicropores (<0.7) and a reduction in the enthalpy of adsorption. Another important task is the development of composite systems based on nanoporous carbon modified with metal nanoparticles, which will enable the implementation of the “spillover” mechanism and increase storage efficiency at temperatures close to room temperature and moderate pressures due to the dissociation of molecular hydrogen and the subsequent migration of atomic hydrogen into the porous matrix. Of significant importance is the development of the concept of programmable pore formation, based on controlling the chemical composition of precursors and the in situ introduction of activating components. This enables control over the formation of a hierarchical porous structure and the surface energy profile. An additional goal is to increase the interaction energy of hydrogen with the surface to a level of approximately 10–15 kJ/mol without significantly degrading the kinetics of adsorption–desorption processes, which requires targeted surface functionalization and optimization of the electronic properties of the carbon matrix. Finally, an important objective is the development of scalable and reproducible technologies for the synthesis of nanoporous carbons from biomass and industrial waste with controlled textural characteristics, which will enable the transition from laboratory research to practical hydrogen storage systems at moderate temperatures and pressures.

## 5. Conclusions

Comparison of current hydrogen storage technologies with the performance targets established by the U.S. Department of Energy (DOE) indicates that no existing storage system simultaneously satisfies all key requirements, including high gravimetric and volumetric storage capacities, rapid charging and discharging kinetics, long-term cycling stability, operational safety, cost-effectiveness, and high system-level energy efficiency. Although compressed and liquefied hydrogen remain the most mature and commercially deployed storage technologies, their widespread application is constrained by high energy consumption, limitations in storage density for mobile applications, and increasingly stringent safety and transportation requirements. Among solid-state storage approaches, metal hydrides and complex hydride systems represent promising alternatives owing to their intrinsically high volumetric hydrogen density and enhanced safety resulting from hydrogen storage in the solid phase. Nevertheless, the practical implementation of most hydride-based materials is hindered by unfavorable thermodynamics, slow hydrogen absorption/desorption kinetics, elevated operating temperatures, and performance degradation during repeated cycling. Consequently, future advances in hydride systems are expected to rely on alloying strategies, catalytic modification, nanostructuring, and the development of composite materials capable of tailoring thermodynamic properties while enhancing hydrogen diffusion and reaction kinetics. In parallel, nanoporous carbon materials derived from renewable biomass have emerged as one of the most promising classes of adsorbents for physical hydrogen storage. Their exceptionally high specific surface area, well-developed microporous architecture, tunable pore-size distribution, and sustainable production routes make them attractive candidates for next-generation storage systems. Recent studies demonstrate that optimization of pore texture together with surface engineering can achieve hydrogen storage capacities exceeding 11 wt.% under cryogenic conditions at moderate pressures, bringing these materials closer than most competing technologies to the US DOE performance targets. Overall, the future development of hydrogen storage technologies will require an integrated multidisciplinary approach combining advances in materials science, nanotechnology, chemical engineering, and computational materials design. Emphasis should be placed on developing materials capable of simultaneously increasing gravimetric and volumetric storage capacities under near-ambient operating conditions, precisely controlling hydrogen binding energies through nanostructuring, defect engineering, and catalytic functionalization, and designing hybrid storage systems that integrate the advantages of adsorption- and hydride-based mechanisms. Furthermore, the integration of first-principles calculations, multiscale modeling, machine learning, and high-throughput materials screening is expected to substantially accelerate the discovery and optimization of next-generation hydrogen storage materials. Taken together, the available evidence suggests that the rational design of nanostructured materials with engineered pore architectures, tailored defect chemistry, catalytic interfaces, and optimized thermodynamic characteristics represents one of the most promising pathways toward hydrogen storage systems capable of approaching the long-term performance targets defined by the US DOE. Continued progress in this direction will be essential for enabling efficient, safe, and economically viable hydrogen storage technologies for large-scale implementation within the emerging hydrogen economy.

## Figures and Tables

**Table 1:** Comparative average characteristics of the three main hydrogen storage approaches based on an analysis of open literature sources.

Storage Method	High-Pressure Cylinders	Cryogenic Tanks	Metal Hydrides (LaNi_5_, TiFe, MgH_2_, etc.)	Nanoporous Materials (Nanoporous Carbon, MOF, COF, Zeolites)
Storage Principle	Hydrogen compression	Cryogenic liquefaction	Chemical adsorption with formation of hydrides	Physical adsorption in micropores
Gravimetric capacity, wt.%	1–6	8–12	0.8–7.6	1–12 (effective at cryogenic temperatures)
Volumetric capacity, kg H_2_/m^3^	20–42	71–87	55–164	15–60
Operating storage pressure	15–70 MPa	0.1–0.5 MPa	0.2–1 MPa	1–10 MPa
Operating storage temperature	−40–85 °C	−253 °C	0–350 °C	−196–24 °C
Heating for hydrogen supply to end-use applications	Not required	Required	25–350 °C	Not required
The need for heat exchange	Not required	Required	Necessarily	Not required
Losses during storage	Minimum	0.2–3% per day	Almost none	Almost none
Refueling speed	High	Medium	Medium	Medium
Delivery speed to the consumer	High	Medium	Medium	Medium
Safety	Danger of storing under high pressure	Danger of evaporation with increasing temperature above cryogenic	High safety due to the bound state of hydrogen	No explosion hazard due to hydrogen dispersion in the microporous structure of the adsorbent

**Table 2:** Comparison of the dehydrogenation (D)/hydrogenation (H) behavior of MgH_2_-based materials doped with different additives.

Composite		Exp. Data				Hydrogenation Capacity, wt.%	Dehydrogenation Capacity, wt.%	Cycle, *n*	Ref.
	Initial *P*, MPa	Heating Treatment, °C/min	Initial *T*, °C *	*T*, °C **	*T*, °C				
MgH_2_-FeTi	1	-	325	265	60	5.4 wt.%	N/A *	N/A *	[78]
MgH_2_-ZrTiO_4_	2.5 D. 0.001 H.	3	249	179	70	5.5 wt.% in 10 min at 125 °C	6.3 wt.% in 5 min at 300 °C	20	[79]
MgH_2_-HfCl_4_	0.1 D. 3 H.	-	410	340	70	5.5 wt.% in 5 min at 300 °C	4.5 wt.% in 5 min at 300 °C	N/A *	[80]
MgH_2_-VNbO_5_	2	5	330	235	95	5 wt.% in 5 min at 160 °C	N/A *	50	[81]
MgH_2_-Ni/CrV-MMO	1–5	5	306	190	116	5.0 wt.% in 10 min at 125 °C	4.3 wt.% in 60 min at 75 °C	20	[82]
MgH_2_-Mo_2_C MXene	2.5	-	342	225	117	6.7 wt.% in 13 min at 300 °C	6.0 wt.% in 12 min at 200 °C	10	[83]
MgH_2_-TiCrNbH_x_-MS	5 R.	1	286	163	123	6.18 wt.% in 12 min at 300 °C	6.34 wt.% in 4 min at 270 °C	10	[84]
MgH_2_-TiNb_2_O_7_			300	177	123	4.5 wt.% in 3 min at 125 °C	5.5 wt.% in 10 min at 250 °C	10	[85]
MgH_2_-Ti-CoO@C	6.5–7	3	346	186	160	5.0 wt.% in 30 min at 150 °C	6.3 wt.% in 5 min at 275 °C	20	[77]

* Onset temperature (°C) for MgH_2_. ** Onset dehydrogenation temperature (°C) for composite. N/A *—not available.

**Table 3:** The influence of FeTi content, as well as additional nickel alloying, on the hydrogen desorption processes of the MgH_2_ + X wt.% FeTi composite.

Composite Composition	Milling Time, h	Temperature *, °C	Temperature **, °C	Decreasing Desorption Temperature, °C	Ref.
MgH_2_ + 10 wt.% FeTi	25	357.6	269.3	88.3	[78]
MgH_2_ + 30 wt.% FeTi	25	333.1	264.8	68.3	
MgH_2_ + 50 wt.% FeTi	50	367.1	365.2	1.9	

* Temperature of the main peak of hydrogen desorption without Ni, ** with Ni.

## Data Availability

The data that supports the findings of this study are available on request from the corresponding author.

## Abbreviations

The following abbreviations are used in this manuscript:

GPHI	Global Programme for Hydrogen in Industry
UNIDO	United Nations Industrial Development Organization
US	United States
DOE	Department of Energy
MOFs	Metal–organic frameworks
DFT	Density functional theory
MD	Molecular dynamics
GCMC	Grand canonical Monte Carlo
FEA	Finite element analysis
SHM	Structural health monitoring
ML	Machine learning
AI	Artificial intelligence
HTS	high-throughput screening
CALPHAD	Calculation of Phase Diagrams
BET	Brunauer–Emmett–Teller
DA	Dubinin–Astakhov
TVFM	Theory of volume filling of micropores
BJH	Barrett–Joyner–Halenda
DR	Dubinin–Radushkevich
HSE	Hydrogen storage enhancement factor
CDCs	Carbide-derived carbons
GHSV	Gas hourly space velocity
WHSV	Weight hourly space velocity
IUPAC	International union of pure and applied chemistry
CFD	Computational fluid dynamics
TEA	Techno-economic analysis
LCA	Life cycle assessment
